# USDA-SoyWheOatBar-3K: a robust high-throughput genotyping platform to accelerate molecular breeding in small grains

**DOI:** 10.3389/fgene.2026.1984445

**Published:** 2026-10-23

**Authors:** Muhammad Massub Tehseen, Mary Osenga, Jared Smith, Amanda J. Waters, Paul St. Amand, Sepehr Mohajeri Naraghi, Kevin P. Smith, Melanie Caffe, Raja Sekhar Nandety, Andrew C. Read, Xuehui Li, Michael S. McMullen, Deven See, Katherine W. Jordan, Gina Brown-Guedira, Jason D. Fiedler

**Affiliations:** 1 Department of Plant Sciences, North Dakota State University, Fargo, ND, United States; 2 USDA-ARS Cereal Crops Improvement Research Unit, Edward T. Schafer Agricultural Research Center, Fargo, ND, United States; 3 USDA-ARS Plant Science Research Unit, Raleigh, NC, United States; 4 Research and Development Division, PepsiCo, Inc., St. Paul, MN, United States; 5 USDA-ARS Hard Winter Wheat Genetics Research Unit, Manhattan, KS, United States; 6 Department of Agronomy and Plant Genetics, University of Minnesota, St. Paul, MN, United States; 7 Department of Agronomy, Horticulture and Plant Science, South Dakota State University, Brookings, SD, United States; 8 USDA-ARS Plant Science Research Unit, St. Paul, MN, United States; 9 USDA-ARS Wheat Health, Genetics, and Quality Research Unit, Pullman, WA, United States

**Keywords:** barley, genomic selection, genotyping, marker-assisted selection, multi-species hybridization, oat, SNP array, wheat

## Abstract

High-throughput genotyping supports marker-assisted selection of known loci and genomic prediction of complex traits. Yet, routine adoption of these molecular breeding tools remains out of reach for many breeding programs. Here, we present a multi-species single-nucleotide polymorphism (SNP) array designed for standardized genotyping of wheat, barley, and oat. The platform contains 9,468 small-grain SNPs (3,421 wheat, 3,058 barley, and 2,989 oat), strategically selected to provide genome-wide coverage using genetic selection bins of 1 cM for wheat and oat and 0.5 cM for barley. Validation across diverse germplasm collections (>10,000 samples) revealed robust technical performance, with 98.7%, 89.0%, and 91.5% of SNPs in barley, wheat, and oat, respectively, meeting all quality thresholds. A useful feature of the array is the ability to genotype samples in multi-species hybridization mode, thereby lowering the array-processing cost per sample. Multi-species hybridization produced partner-specific signal responses while retaining high genotype-call concordance, ranging from 93.5% to 98.7% across the evaluated dual- and tri-hybridization configurations. Oat signal quality was highest under tri-hybridization, although its performance was only marginally greater than in the oat-wheat dual configuration. Independent applications demonstrated that the array produced genomic prediction accuracies comparable to those obtained using higher-density arrays or sequence-based genotyping platforms and supported the detection of marker-trait associations in genome-wide association studies. By combining species-specific marker sets for wheat, barley, and oat on one platform, the array provides a standardized genomic resource for routine molecular breeding.

## Introduction

Small-grain cereal crops, including wheat (*Triticum aestivum*), barley (*Hordeum vulgare*), and oat (*Avena sativa*), play a crucial role in global food security, contributing 25% of human caloric intake worldwide (Awika, 2011). In the face of increasing demand, emerging diseases, and variable growing conditions, the improvement of small grains has intensified in regionally focused breeding programs (Poland et al., 2012). One of the tools available to increase the efficiency of genetic gain and accelerate the release of new varieties is the use of genomic information to predict performance; enabling selection of lines with desirable traits - or rejection of those with undesirable traits - early in the breeding cycle (Rasheed et al., 2017).

High-throughput genotyping platforms are instrumental in unlocking the genome for crop improvement via molecular breeding. Genome-wide polymorphism data is used to identify genetic variations across large populations, which is critical for applying advanced genomics-based methods such as genome-wide association studies (GWAS) and quantitative trait loci (QTL) mapping to discover trait-linked molecular markers that influence traits. These markers are then converted into high-throughput targeted assays such as allele-specific PCR to screen breeding germplasm for the favorable alleles in marker-assisted selection (MAS) pipelines (Ali et al., 2025; Kaur et al., 2020). In addition, genome-wide platforms can be used directly in genomic selection (GS) breeding designs to estimate breeding values for all traits simultaneously. Implementing these molecular tools with greater selection stringency and/or larger numbers of early-generation lines offers promising approaches to accelerate genetic gain (Crossa et al., 2017). Among the various genome-wide genotyping technologies available, Genotyping-by-Sequencing (GBS), whole genome sequencing (WGS), capture-based and amplicon sequencing approaches, as well as SNP arrays, are particularly noteworthy due to their wide applicability in small grains breeding.

Sequencing-based genotyping approaches including whole-genome sequencing, genotyping-by-sequencing (GBS), sequence capture, and multiplexed amplicon sequencing have greatly expanded variant discovery in small grains. Whole-genome sequencing provides near-saturating marker density and has supported gene discovery and cloning, but it remains costly for routine genotyping of large breeding populations with complex genomes (Athiyannan et al., 2022; Bevan and Uauy, 2013; Chai et al., 2018; Close et al., 2009; Schreiber et al., 2018). GBS and related reduced-representation methods lower per-sample cost and have been widely applied in wheat, barley, and oat (Akram et al., 2021; Arruda et al., 2016; Darrier et al., 2019; Elshire et al., 2011; Klos et al., 2017; Yan et al., 2023), but variable locus recovery, missing data, and dependence on bioinformatic processing can complicate comparisons across projects, populations, and breeding cycles (Voss-Fels and Snowdon, 2016). Capture- and amplicon-based approaches improve consistency at predefined targets and have enabled high-resolution genotyping and gene discovery in small grains (Bernardo et al., 2020; Dreisigacker et al., 2026; Mascher et al., 2013; Pankin et al., 2014; Ruff et al., 2020; Steuernagel et al., 2016), although their cost and effective marker density remain dependent on DNA quality, assay design and sequencing depth. In contrast, SNP arrays interrogate a fixed set of validated loci and provide standardized genotype calls, high reproducibility, rapid processing, and direct comparability across laboratories, populations, and breeding cycles (Thomson, 2014; Wang et al., 2014). These characteristics make arrays particularly suitable for routine genomic selection and marker-assisted breeding, provided that marker density, probe specificity, and ascertainment are optimized for the target germplasm (Burridge et al., 2024; Wang et al., 2014). Among fixed-locus genotyping platforms, the Illumina Infinium II iSelect and Thermo Fisher Axiom products have been widely used in crop genetics and breeding. These assays offer high reproducibility, data coverage, scalability, and quick generation of ready-to-use genotype calls for large sample numbers but require prior knowledge of the genome for probe design (Wang et al., 2014).

For barley, the evolution of SNP arrays includes the 1,572-SNP Illumina GoldenGate assay introduced in 2006 (Close et al., 2009), the Illumina Infinium iSelect 9K SNP array (Comadran et al., 2012), and the Illumina 50K array featuring over 43,000 scorable SNPs derived from exome capture data and backward-compatible markers from the earlier 9K array (Bayer et al., 2017). Additionally, the Illumina Wheat Barley 40K SNP array, a multi-species platform, contains 14,261 barley-specific SNPs, allowing joint hybridization of wheat and barley samples in the same assay (Keeble-Gagnère et al., 2021).

In oat, the progression of genotyping arrays includes the initial Illumina 3,072 SNP array (Oliver et al., 2013), the 6 K array facilitating genotyping of diverse populations (Tinker et al., 2014), and the 10 K oat array, an expanded version of the 6 K platform, providing higher marker density for improved genomic selection and trait mapping applications (Chaffin et al., 2016). These SNP arrays collectively represent powerful tools for genomic studies and breeding programs across small grains, offering various marker densities and applications in research and breeding.

In wheat, several SNP arrays have been developed, including the Illumina 9K array (Cavanagh et al., 2013), the 15K SNP array (Boeven et al., 2016), the 90K array (Wang et al., 2014), the Axiom 35K Wheat Breeder's genotyping array (Allen et al., 2017), the Axiom 820K Wheat HD SNP array (Winfield et al., 2016), and the recently developed *Triticum aestivum* Next Generation (TaNG) array containing over 43,000 SNPs optimized for varietal discrimination and GWAS applications (Burridge et al., 2024). These arrays have facilitated many genome-wide association studies (GWAS) and marker-assisted selection in breeding programs.

Despite the availability of diverse genotyping platforms, there remains a need for new standardized, rapid, high-throughput platforms tailored to routine small-grain breeding. Fixed-locus arrays provide consistent marker recovery across germplasm sets and successive breeding generations, however, high-density arrays may provide more resolution than is required for routine selection at a relatively high price-point (Thomson, 2014; Voss-Fels and Snowdon, 2016). On the other hand, declining sequencing costs make GBS relatively inexpensive, with the trade-off of increased bioinformatic requirements and variable locus recovery every year. Mid-density, fixed-content platforms have accordingly been advanced as a cost-effective compromise for operational genomic selection, with public mid-density panels now recently being deployed at scale in wheat breeding programs (Dreisigacker et al., 2026).

To address this gap, we developed a multi-species, genome-wide genotyping platform based on Illumina Infinium II array technology. The platform targets approximately 3,000 strategically selected SNPs in each of wheat, oat, and barley, providing a fixed marker density suited to routine breeding applications.

## Materials and methods

### SNP discovery

SNP discovery was conducted across three small grain species, wheat, barley, and oat, using species-specific strategies and reference genomes to ensure high-quality and informative markers. The wheat discovery panel comprised 384 lines representing five regional breeding groups, Central, Northern, Eastern, Pacific Northwest spring, and Pacific Northwest winter wheat. Exome-capture sequencing was conducted using a previously published method (Jordan et al., 2022). Reads were aligned to the IWGSC Chinese Spring RefSeq v2.1 reference genome (Zhu et al., 2021) using Bowtie 2 (Langmead and Salzberg, 2012). Variant calling was conducted with BCFtools and SAMtools (Danecek et al., 2021) using properly paired reads with mapping quality >20, followed by stringent filtering based on per-genotype read depth, SNP coverage, SNP quality, and overall minor allele frequency (MAF). A total of 284,657 high-confidence SNPs remained after filtering for the genome-wide selection process. Additionally, 350 candidate SNPs associated with morpho-physiological traits and disease resistance were evaluated for potential inclusion as MAS assays. For barley, SNP discovery leveraged the large amount of historical breeding germplasm genotyping data from the 50K iSelect SNP array (Bayer et al., 2017) previously generated at the North Central Small Grains Regional Genotyping Laboratory. SNP information from the legacy 9K array was also included to maintain backward compatibility (Comadran et al., 2012). Genotypes from 13,571 samples assayed with the 50K array and 3,760 samples assayed with the 9K array informed genome-wide SNP selection. A set of 207 candidate trait-associated QTL and SNP markers linked to agronomic traits and disease resistance was evaluated for potential inclusion as MAS assays. Sequences flanking the array SNPs were aligned to the Morex v3 reference genome (Mascher et al., 2021) for accurate positioning.

In oat, genotyping data from 7,353 samples from the Illumina 6K array (Tinker et al., 2014) were combined with another 1,166 breeding lines with genome-wide sequencing data. Positions for all SNPs were lifted to the OT3098 v2 reference genome (NCBI assembly GCA_916181665.1; National Center for Biotechnology Information, 2022). Additionally, 1,730 well-performing segregating markers from a proprietary PepsiCo Illumina oat array were provided to assist in genome gap filling and 283 candidate trait-associated markers were evaluated for potential inclusion as MAS assays. Following probe-design and technical evaluation, the complete marker inventory and associated metadata are provided in Supplementary Table S1. Trait-linked marker information for the 490 assays designated for MAS, comprising 231 wheat, 94 barley, and 165 oat SNPs, is provided in Supplementary Table S2.

### Selection of SNP positions and probes for the genotyping assay

The SNP selection process focused on achieving uniform genomic coverage while prioritizing markers informative across diverse germplasm, with a specific focus on usefulness within individual breeding programs. For all species, genotyping data were filtered and processed using standardized criteria. Markers with MAF below 0.05, call rates below 95% for array data or 50% for sequencing data, or equally good alignments to multiple reference-genome positions were excluded (Anderson et al., 2010). Principal component analysis and cluster analysis/designation were conducted with mclust version 6.1.1 (Scrucca et al., 2016) in R version 4.4.1 (R Core Team, 2024) to identify population structure with the combined input genotyping data sets. Minor allele frequency was calculated for each sub-cluster individually. Published consensus linkage maps were used to estimate the approximate genetic bin location of all the SNPs based on a common reference genome alignment (Bayer et al., 2017; Chaffin et al., 2016; Wang et al., 2014). Within genetic bins of 1 cM (wheat and oat) and 0.5 cM (barley), a single marker was selected after ranking based on MAF, the number of sub-clusters with MAF >0.05, association with a trait, presence on previous platforms, and array cluster separation (if available). The smaller bin size used for barley reflects its substantially shorter seven-chromosome genetic map relative to the 21-chromosome wheat and oat maps; using 0.5-cM intervals yielded a number of candidate selection intervals more comparable to the approximately 3,000-marker target shared across crops. Gaps in the genome were filled in with additional selections based on physical intervals. All SNPs associated with a trait were also added if they were not previously selected. Selected SNPs were submitted to the Illumina Assay Design Tool to predict assay performance and probe design. Failed markers were replaced with the second-best marker within that genetic interval. To further refine the assay design, cross-hybridization potential was assessed by aligning probe sequences against reference genome assemblies, ensuring probes predominantly targeted low-copy genomic regions. In some cases, a manual probe sequence corresponding to the opposite flanking sequence was designed to limit multiple-hybridization events. This step was critical to minimize ambiguous signals arising from duplicated loci, particularly in polyploid species like wheat and oat. The final array design incorporated a combination of Infinium I and Infinium II assays, tailored to the specific needs of the targeted small grain species.

The final manufactured small-grain component contained 9,468 SNPs, comprising 3,421 wheat, 3,058 barley, and 2,989 oat SNPs. These markers were combined with 3,212 soybean markers already in production (Song et al., 2024), yielding the USDA-SoyWheOatBar-3K array (Supplementary Table S1). The present study evaluates the newly added small-grain component and hybridizations among wheat, barley, and oat; soybean-containing hybridizations were outside the scope of this study. The platform supports genome-wide association studies (GWAS) (Tibbs Cortes et al., 2021), genomic selection (GS) (Meuwissen et al., 2001), and marker-assisted selection (MAS) (Collard and Mackill, 2008).

### Genotyping and data processing

Validation panels comprised 5,046 wheat, 2,301 barley, and 2,675 oat samples submitted by 22 U.S. breeding programs—six wheat, nine barley, and seven oat programs—and associated diversity panels. Samples were processed at the North Central Regional Small Grains Genotyping Laboratory in Fargo, ND. Each DNA sample (∼200 ng) was genotyped following standard Illumina Infinium HTS/XT protocols, and fluorescence intensities were scanned using the Illumina iScan system. In addition to the standard single-species protocol, selected samples were genotyped under dual- and tri-hybridization modes to evaluate assay performance across different configurations. In these modes, DNA was pooled at species-specific mass ratios intended to approximate equal numbers of haploid genome equivalents based on the relative genome sizes of wheat, oat, and barley (Kamal et al., 2022; Mascher et al., 2021; Zhu et al., 2021), rather than at equal DNA mass, before processing with the Infinium protocol. Wheat/barley pools consisted of 150 ng and 50 ng of DNA, respectively. Oat/barley pools consisted of 150 ng and 75 ng of DNA, respectively. Wheat/oat pools consisted of 125 ng and 75 ng of DNA, respectively. Finally, wheat/oat/barley pools consisted of 170 ng, 120 ng, and 50 ng of DNA, respectively. The corresponding wheat:barley, oat:barley, wheat:oat, and wheat:oat:barley DNA mass ratios were 3:1, 2:1, 1.67:1, and 3.4:2.4:1, respectively.

SNP clustering and genotype calling operations were carried out using GenomeStudio Polyploid Genotyping Module (version 2.0.5; Illumina, Inc.). Species-specific cluster files were generated with the validation panels, using the reference sequence-based genotype calls to guide ambiguous cluster assignments when available.

### SNP categorization analysis

SNP data analysis was performed using R version 4.1.0 (R Core Team, 2021) with packages tidyverse (v1.3.1) (Wickham et al., 2019), dplyr (v1.0.7) (Wickham et al., 2021), ggplot2 (v3.3.5) (Wickham, 2016), and viridis (v0.6.2) (Garnier et al., 2021) for data manipulation and visualization. For quality assessment, SNPs were evaluated using multiple criteria. Per-sample genotype calls (AA, AB, BB), normalized signal intensity (R), and normalized allelic intensity ratio (Theta) values were extracted from the GenomeStudio project full-data table. Theta and R are plotted on the x- and y-axes, respectively, in two-dimensional cluster plots used to infer genotype clusters and assign genotype calls for each sample at a given marker. No calls (NCs) were defined as observations with fluorescent signal that could not be assigned unambiguously to an AA, AB, or BB cluster. NULL calls were defined as observations with little or no target amplification. NC and NULL frequencies were calculated and reported separately because NC represents an ambiguous genotype assignment, whereas a reproducible NULL state may reflect an additional absence-of-amplification allele. SNPs were flagged as problematic if they exceeded thresholds for no-call and null-call (no hybridization) rates (>20%), showed high within-cluster deviation in Theta (>0.20) or R (>1.0) values, exhibited poor cluster separation (<0.1 Theta between adjacent clusters), or displayed excessive heterozygosity (>50%) in the validation panels. Based on these criteria, SNPs were classified as “Pass” or assigned specific quality issue designations.

Interaction and competition of similar genetic sequences from the two or more different crops at a given probe can result in a shifting of the Theta and R values, and subsequent changes in genotype calling. For multi-species hybridization comparisons (dual and tri), we evaluated concordance across assay configurations using genotype calls from single-species mode as the reference. The process began by removing NC genotypes and filtering outliers based on IQR criteria using a 1.5×IQR multiplier beyond the first (25th percentile) and third (75th percentile) quartiles (Páez and Boisjoly, 2022). For each SNP, the mean absolute Theta shift was calculated as Δθ = n^−1^Σ|θ_single - θ_multi| across retained matched observations, and Theta correlation (rθ) was calculated between the single- and multi-species configurations. The composite quality score was then calculated as Q = (1 - Δθ) × rθ. SNPs were classified as Excellent (Q ≥ 0.90), Good (0.80 ≤ Q < 0.90), Fair (0.70 ≤ Q < 0.80), or Poor (Q < 0.70).

Theta shift analysis characterized the magnitude and direction of signal changes between hybridization modes, with summary statistics calculated for each genotype cluster. A balanced SNP subset analysis identified markers with adequate representation across genotype classes, highlighting top and bottom performers based on quality scores.

Signal-quality analyses were conducted using the IQR-filtered Theta observations, whereas genotype-call concordance was calculated from all matched genotype calls without applying the IQR filter. Observations with missing genotype calls in either the single- or multi-species configuration were excluded from the genotype-call comparison.

Genotype calls from matched samples were compared after excluding observations that were missing in either dataset. Concordance was calculated as $C=\left(1/N\right)\sum_{i=1}^{N}I\left(x_{i}=y_{i}\right)$ , where N is the number of non-missing matched calls and I is an indicator function. The miscall rate was calculated as 1−C . Pearson correlation was calculated after coding AA, AB, and BB as 0, 0.5, and 1, respectively. Cohen’s kappa was calculated as $\kappa =\left(p_{o}-p_{e}\right)/\left(1-p_{e}\right)$ , where $p_{o}$ is the observed call agreement and $p_{e}$ is the agreement expected from the marginal genotype frequencies.

## Results

### Development and design of the USDA-SoyWheOatBar-3K genotyping array

The small-grain component of the USDA-SoyWheOatBar-3K array was developed to provide mid-density, species-specific SNP coverage for wheat, barley, and oat, with approximately 3,000 SNPs selected for each species. The final small-grain component contained 9,468 SNPs, comprising 3,421 wheat, 3,058 barley, and 2,989 oat SNPs. The final marker inventory classified 490 assays as MAS markers: 231 wheat, 94 barley, and 165 oat SNPs (Supplementary Table S2).

Of the 3,421 wheat SNPs, 3,356 were derived from sequencing and 65 from the Wheat 90K array. Based on their intended purpose, 3,188 were genome-wide tagging markers and 231 were designated MAS markers; two additional intended MAS assays failed technical evaluation. These markers were distributed across all 21 wheat chromosomes, with greater marker density in the A and B subgenomes than in the D subgenome (Figure 1).

**FIGURE 1:**
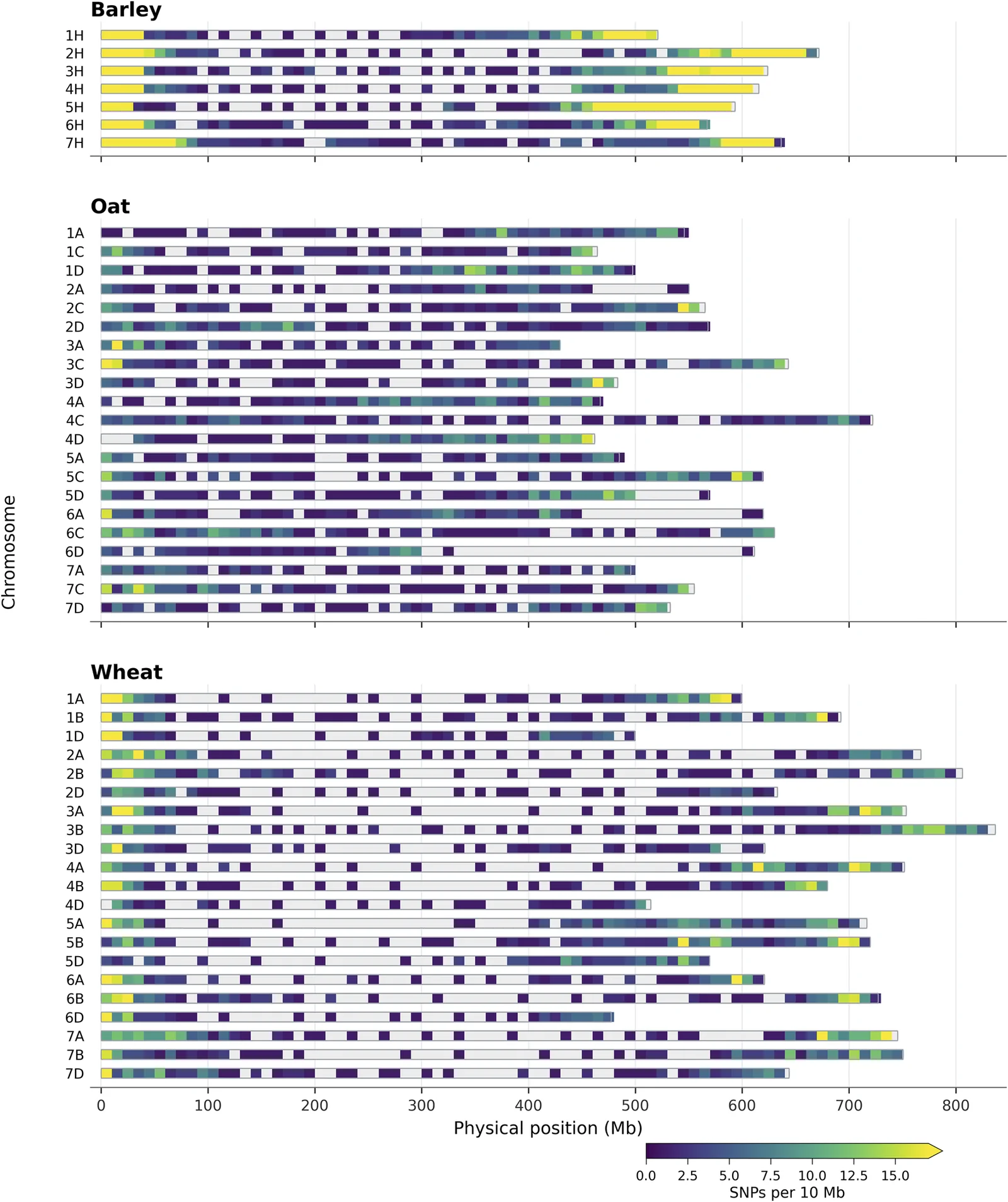
Chromosomal distribution of SNPs in barley, oat, and wheat included in the USDA-SoyWheOatBar-3K.

For barley, of the 3,058 SNPs, 2,745 were derived from the Barley 50K array (Bayer et al., 2017), 282 from the legacy 9K array, 30 from sequencing, and one from a cloned assay. Based on their intended purpose, 2,963 were genome-wide tagging markers and 94 were designated MAS markers. One additional intended MAS assay failed technical evaluation. Markers were distributed across the Morex v3 reference genome, with higher densities in the more recombinogenic distal chromosome regions.

Of the 2,989 oat SNPs, 942 were derived from the Oat 6K array, 930 from the proprietary Infinium array, and 1,117 from sequencing. Based on their intended purpose, 2,824 were genome-wide tagging markers and 165 were designated MAS markers. SNPs were positioned on the OT3098 v2 and Sang v1 reference genomes (Kamal et al., 2022) to support placement across the 21 oat chromosomes (Figure 1).

Across all three species, the filtering pipeline effectively reduced large initial SNP pools to high confidence sets suitable for array design. The selected SNPs exhibited minimal missing data, high call rates, and polymorphism across diverse germplasm, ensuring robust performance in downstream genotyping applications. These curated SNP sets formed the foundation for the USDA-SoyWheOatBar-3K array, supporting standardized high-throughput genotyping for genomic analyses in wheat, barley, and oat.

### Data quality assessment

The performance of the USDA-SoyWheOatBar-3K genotyping array was evaluated using extensive validation panels comprising 5,046 wheat, 2,301 barley, and 2,675 oat samples, representing diverse germplasm collections including several independent breeding programs. Standardized quality metrics were applied across all three species.

Technical performance of each SNP cluster was evaluated across several metrics including overall fluorescent signals (R), variation in the theta and R directions, average separation from each other and presence of a third “heterozygous” genotype cluster. Probes displaying inferior metrics in these qualities resulted in inaccurate genotype calling and/or high levels of missing data and were marked as problematic. Overall performance was very good among this set of markers. Barley SNPs demonstrated the highest technical quality, with only 1.3% (41/3,058) classified as problematic (Table 1). In contrast, wheat and oat exhibited higher proportions of problematic markers at 11.0% (377/3,421) and 8.5% (255/2,989), respectively. This pattern aligns with the relative genome complexities of these species, with the hexaploid wheat and oat genomes presenting greater challenges for robust SNP performance compared to the diploid barley genome.

**TABLE 1:** Summary of SNP genotyping quality assessment across species.

Species	Sample(n)	SNPs	Problematic SNPs (%)	NC/NULL issues	Theta var (AA/AB/BB)	R var (AA/AB/BB)	Cluster issues	Het issues
Barley	2,301	3,058	41 (1.3%)	1/16	0/2/0	0/0/0	1	21
Oat	2,675	2,989	255 (8.5%)	32/69	8/14/3	0/0/3	13	123
Wheat	5,046	3,421	377 (11.0%)	173/115	3/1/11	0/0/0	63	17

* Quality-issue categories are not mutually exclusive; therefore, the sum of the category counts may exceed the total number of problematic SNPs.

The most prevalent quality issues were different across species. In barley, heterozygosity issues dominated (21/41, 51% of problematic SNPs), while in oat, heterozygosity issues (123/255, 48%) and NC/NULL call issues (101/255, 40%) were most common. For wheat, NC/NULL call issues were predominant (288/377, 76%), followed by cluster separation issues (63/377, 17%). These patterns reflect the specific technical challenges associated with each genome’s characteristics.

Call rate performance varied by species, with wheat showing the highest proportion of no-call issues (5.1% of SNPs), compared to oat (1.1%) and barley (0.03%). This pattern was consistent with null-call rates, where 3.4%, 2.3%, and 0.5% of SNPs were affected in wheat, oat, and barley, respectively. The superior call-rate performance of barley SNPs again reflects the relative simplicity of the diploid barley genome compared to the more complex polyploid wheat and oat genomes.

Representative cluster plots clearly illustrate the species-specific SNP quality challenges (Figure 2). The wheat SNP (ExcapSNP-3A_187) exhibits high theta variation across the BB genotype cluster, the oat SNP (Avyb_95344) shows pronounced heterozygosity issues with poorly defined clusters, and the barley SNP (VrnH2_9) demonstrates high null call rates that result in an additional signal cluster outside the expected pattern. These visual patterns are consistent with the quantitative metrics and highlight the species-specific technical challenges encountered in array development.

**FIGURE 2:**
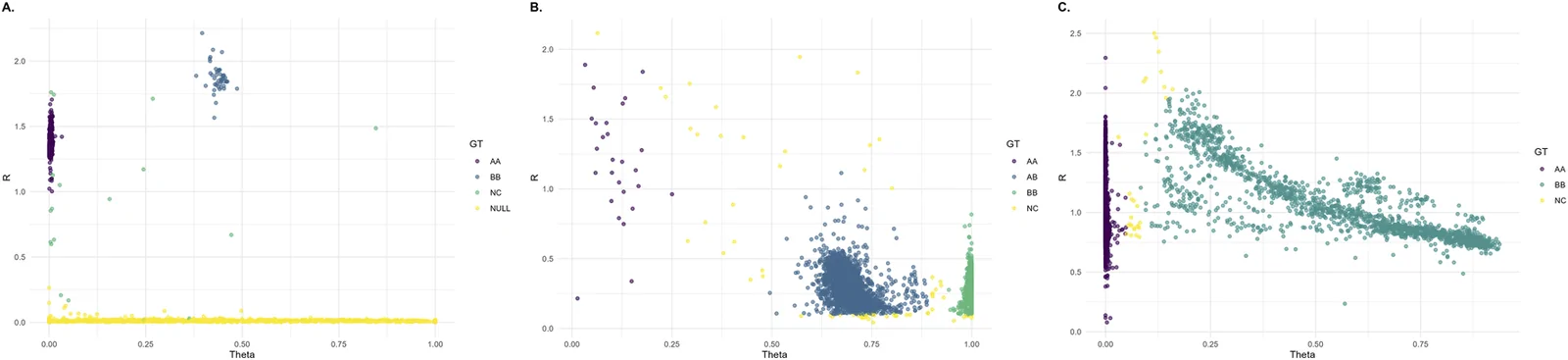
Representative problematic SNP cluster patterns identified during quality control of the USDA-SoyWheOatBar-3K array: **(A)** barley marker VrnH2_9, showing an elevated NULL-call rate; **(B)** oat marker Avyb_95344, showing excessive apparent heterozygosity and poorly resolved clusters; and **(C)** wheat marker ExcapSNP-3A_187, showing elevated within-cluster Theta variation in the BB genotype class.

Overall, the USDA-SoyWheOatBar-3K array demonstrated robust technical performance, with 98.7%, 89.0%, and 91.5% of SNPs in barley, wheat, and oat, respectively, meeting all quality thresholds. The observed quality differences between species were consistent with expectations based on their genomic characteristics and highlight the particular challenges in developing high-performance SNP assays for complex polyploid genomes.

### Genotyping performance

To assess concordance with the source datasets, genotype calls from samples assayed with the USDA-SoyWheOatBar-3K array were compared with matched source-platform genotypes.

Source-platform comparisons included Barley 50K versus USDA-3K for barley, exome-capture sequencing versus USDA-3K for wheat, and resequencing, Oat 6K, and GBS datasets versus USDA-3K for oat.

Under single-species hybridization, homozygous genotype calls from the USDA-SoyWheOatBar-3K array showed 97.6%, 95.0%, and 92.1% concordance with the source-platform genotypes in wheat, barley, and oat, respectively. Including heterozygotes consistently reduced concordance and r^2^. Concordance was stable across MAF in wheat, but declined below 5% MAF in oat, while barley performed best at low MAF (Figure 3).

**FIGURE 3:**
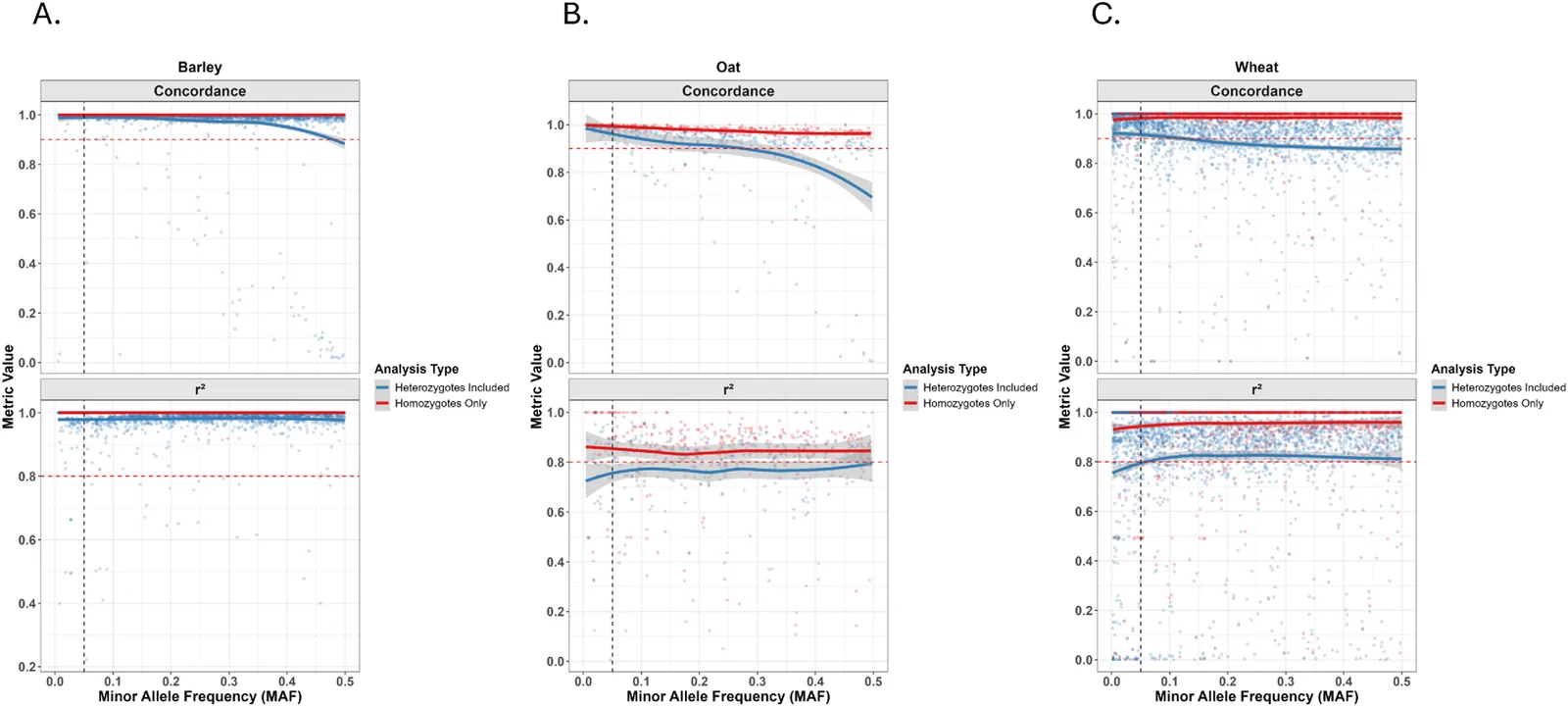
Concordance and r^2^ across MAF bins for selected barley, oat and wheat dataset comparisons between 3K array and corresponding original platforms for **(A)** barley, **(B)** oat, and **(C)** wheat.

In addition to showing concordance with the source datasets, the array retained useful within-program polymorphism. Among the 22 U.S. small-grain breeding programs represented in the initial validation panels—seven oat, nine barley, and six wheat programs—at least 2,000 markers segregated at MAF ≥0.05 in every program except the durum wheat program.

### Multi-species hybridization performance

Multi-species configurations were evaluated relative to the corresponding single-species reference mode, with each dual-species partner analyzed separately (Table 2; Figure 4). For barley SNPs, signal quality was highest in the barley-oat dual configuration, with a mean composite quality score of 0.9049 Â ± 0.2781 and 89% of SNPs classified as Excellent. Barley-wheat dual hybridization produced a mean quality score of 0.8021 Â ± 0.3870 and 78.45% Excellent SNPs, whereas barley tri-hybridization produced the lowest signal-quality performance, with a mean score of 0.6676 Â ± 0.4826 and 64.95% Excellent SNPs.

**TABLE 2:** Partner-specific signal quality and genotype-call agreement under dual- and tri-hybridization relative to single-species mode.

Crop	Configuration	SNPs	Quality score, mean ± SD	Quality categories, E/G/F/P, n (%)	Mean |Theta shift|, AA/AB/BB	Concordance (%)	Pearson r	Cohen’s κ	Miscall rate within category, E/P (%)
Barley	Barley-oat dual	3,052	0.9049 ± 0.2781	2,725 (89.29)/23 (0.75)/10 (0.33)/294 (9.63)	0.0067/0.0324/0.0068	98.71	0.989	0.975	1.03/3.32
Barley	Barley-wheat dual	3,054	0.8021 ± 0.3870	2,396 (78.45)/48 (1.57)/21 (0.69)/589 (19.29)	0.0077/0.0454/0.0084	94.24	0.922	0.888	5.75/5.09
Barley	Tri-hybridization	2,964	0.6676 ± 0.4826	1,925 (64.95)/25 (0.84)/10 (0.34)/1,004 (33.87)	0.0065/0.0376/0.0070	96.68	0.961	0.933	2.67/4.45
Oat	Oat-barley dual	2,935	0.8268 ± 0.3022	2,063 (70.29)/286 (9.74)/87 (2.96)/499 (17.00)	0.0279/0.0564/0.0183	97.21	0.978	0.948	2.05/2.06
Oat	Oat-wheat dual	2,933	0.8863 ± 0.2383	2,347 (80.02)/220 (7.50)/59 (2.01)/307 (10.47)	0.0237/0.0517/0.0170	97.71	0.984	0.957	1.55/3.00
Oat	Tri-hybridization	2,928	0.8926 ± 0.2261	2,372 (81.01)/208 (7.10)/57 (1.95)/291 (9.94)	0.0230/0.0481/0.0157	97.76	0.985	0.958	1.47/3.22
Wheat	Wheat-barley dual	3,260	0.8250 ± 0.2976	2,278 (69.88)/276 (8.47)/128 (3.93)/578 (17.73)	0.0284/0.0465/0.0205	98.44	0.978	0.969	1.41/0.88
Wheat	Wheat-oat dual	3,377	0.6877 ± 0.4078	1,863 (55.17)/277 (8.20)/112 (3.32)/1,125 (33.31)	0.0362/0.0712/0.0283	93.51	0.928	0.878	5.07/5.96
Wheat	Tri-hybridization	3,379	0.6970 ± 0.3971	1,879 (55.61)/281 (8.32)/133 (3.94)/1,086 (32.14)	0.0382/0.0707/0.0303	94.82	0.922	0.898	4.05/4.56

E, Excellent; G, Good; F, Fair; P, Poor. Quality categories are presented as the number and percentage of analyzed SNPs. Mean absolute Theta shifts are shown in the order AA/AB/BB. Genotype concordance and category-specific miscall rates were calculated across matched observations with non-missing genotype calls in both the single- and multi-species configurations. The single-species genotype call was treated as the reference. Genotype miscall rate was calculated as 1 - genotype concordance among matched non-missing calls within each SNP quality category. Pearson correlation was calculated after coding AA, AB, and BB as 0, 0.5, and 1, respectively. Cohen’s κ represents agreement between single- and multi-species genotype calls after accounting for agreement expected from marginal genotype frequencies.

**FIGURE 4:**
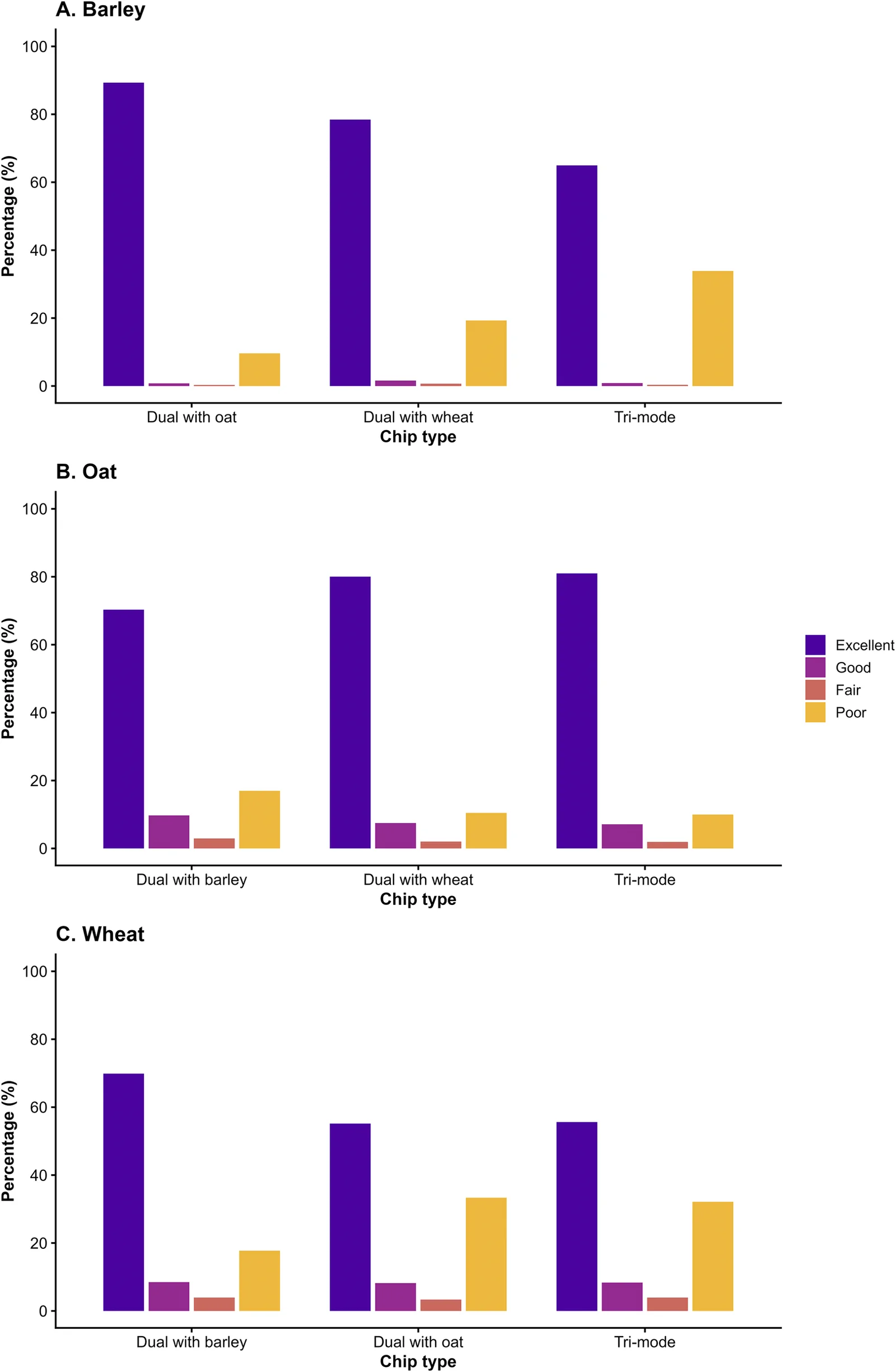
Distribution of SNP signal-quality categories for barley **(A)**, oat **(B)**, and wheat **(C)** under the evaluated dual- and tri-hybridization configurations. Quality categories were assigned from the composite score combining mean absolute Theta shift and Theta correlation relative to the corresponding single-species reference mode.

Oat SNP performance was lowest in the oat-barley dual configuration, with a mean quality score of 0.8268 Â ± 0.3022 and 70.29% Excellent SNPs. Oat-wheat dual hybridization produced a mean quality score of 0.8863 Â ± 0.2383 and 80.02% Excellent SNPs. Tri-hybridization was marginally higher, with a mean quality score of 0.8926 Â ± 0.2261 and 81.01% Excellent SNPs.

Wheat SNPs performed best in the wheat-barley dual configuration, with a mean quality score of 0.8250 Â ± 0.2976 and 69.88% Excellent SNPs. Wheat-oat dual hybridization and wheat tri-hybridization produced similar but lower signal-quality performance, with mean quality scores of 0.6877 ± 0.4078 and 0.6970 ± 0.3971, respectively, and 55.17% and 55.61% Excellent SNPs. Across all configurations, the AB genotype class generally exhibited larger absolute Theta shifts than either AA or BB, indicating that heterozygous clusters were more sensitive to changes in hybridization conditions.

### Genotype concordance

Genotype-call agreement remained high despite the partner-specific differences observed in signal-quality metrics. Across the nine crop-by-hybridization comparisons, genotype concordance ranged from 93.51% to 98.71%, Pearson correlations ranged from 0.922 to 0.989, and Cohen’s κ values ranged from 0.878 to 0.975 (Table 2). For barley, genotype concordance was 98.71% in barley-oat dual hybridization, 94.24% in barley-wheat dual hybridization, and 96.68% under tri-hybridization. Oat concordance was 97.21%, 97.71%, and 97.76% in oat-barley dual, oat-wheat dual, and tri-hybridization, respectively. Wheat concordance was 98.44% in wheat-barley dual hybridization, 93.51% in wheat-oat dual hybridization, and 94.82% under tri-hybridization. Poor signal-quality SNPs generally had higher miscall rates than Excellent SNPs, but this relationship was not universal. Poor SNPs retained genotype concordance ranging from 94.04% to 99.12%, and their miscall rates were lower than those of Excellent SNPs in the barley-wheat and wheat-barley configurations. These results demonstrate that movement or dispersion of the fluorescence clusters did not necessarily result in a mis-identified genotype call.

## Case studies: performance of the USDA-SoyWheOatBar-3K SNP array in genomic selection and GWAS

### Genomic selection: prediction accuracy across small grains

Studies implementing the USDA-SoyWheOatBar-3K SNP array in genomic selection demonstrated its effectiveness and cost efficiency in oat breeding programs. Bazzer et al. (2025) evaluated beta-glucan content in 1,230 breeding lines over 7 years in the South Dakota State University oat program and found that the array achieved similar prediction accuracy compared to genotyping-by-sequencing (GBS) despite having fewer markers, making it a promising platform for implementing genomic selection.

In another report, Dhakal et al. (2024) successfully implemented multi-trait genomic selection (MTGS) using the GBS and USDA-3K array data for improving grain milling quality traits with 558 breeding lines in the University of Illinois oat breeding programs. The research demonstrated that MTGS models incorporating kernel morphometric traits as secondary traits achieved substantial improvements over single-trait genomic selection, with prediction accuracies reaching 0.59 for test weight, 0.60 for thins percentage, and 0.47 for groat percentage. Notably, the best-performing multi-trait cross-validation scheme (MT-CV2) that included kernel morphometric traits in both training and candidate sets outperformed single-trait models by 52% for test weight and 59% for thins percentage.

In wheat, Brault et al. (2025) applied genomic prediction for Fusarium head blight (FHB) resistance in the USDA hard red spring wheat uniform nursery, reporting prediction accuracies ranging from 0.49 to 0.72 across different models and traits. The USDA-SoyWheOatBar-3K array predictors performed equivalently to the 90K markers and the highest accuracy was achieved using reproducing kernel Hilbert space (RKHS) models, demonstrating the array’s suitability for predicting complex disease resistance traits (Figure 5). Additionally, the USDA-SoyWheOatBar-3K was used to characterize and track the Rht gene(s) distribution of historical entries to this nursery (Gill et al., 2026).

**FIGURE 5:**
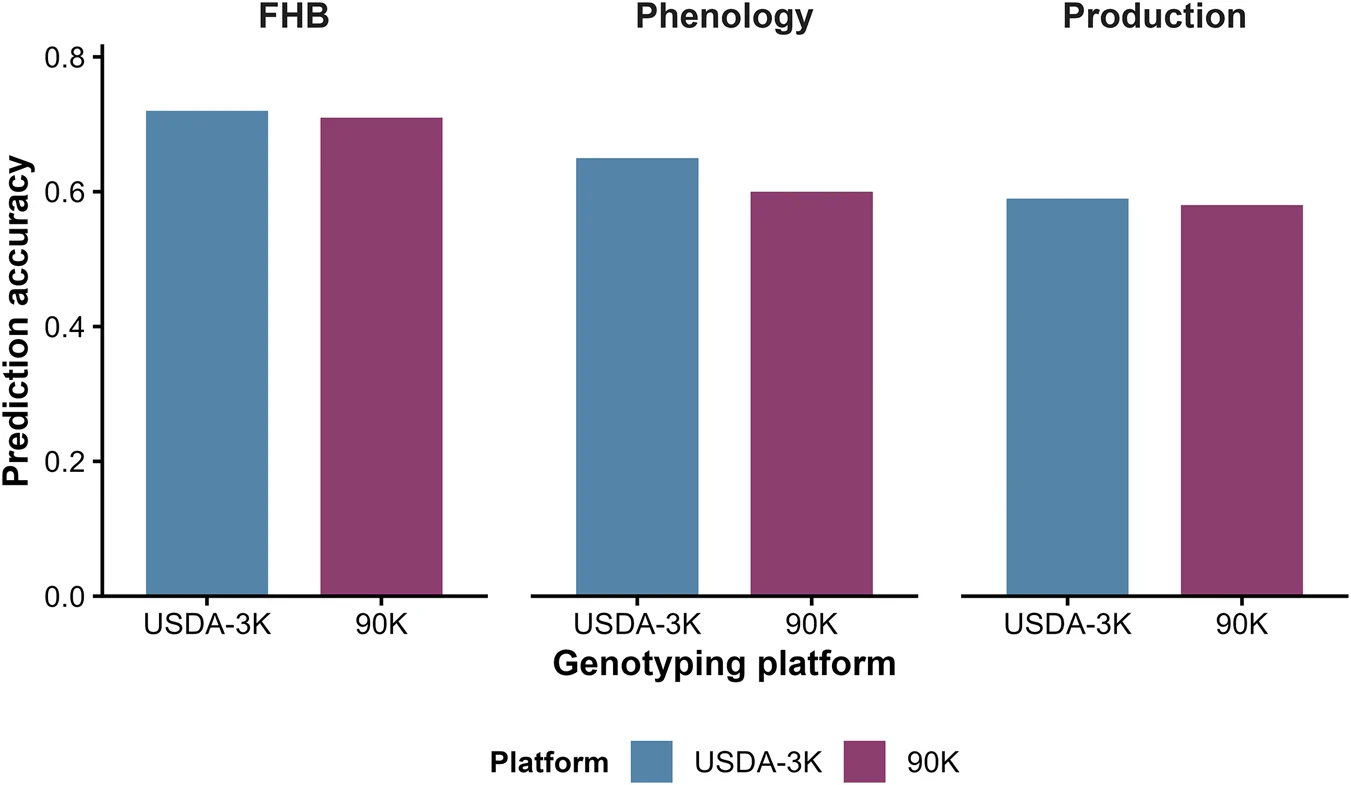
Genomic prediction accuracy comparison between USDA-SoyWheOatBar-3K and high-density 90K array across trait categories in wheat.

### Genome-wide association studies: marker-trait associations

The USDA-SoyWheOatBar-3K SNP array has proven effective for GWAS in small-grain breeding programs. By providing well-spaced, high-confidence SNP data, the platform enables the detection of marker-trait associations critical for trait dissection and molecular breeding applications.

Bazzer et al. (2025) applied the 3K array to dissect the genetic basis of β-glucan content in oats. Their multi-environment GWAS identified 73 significant loci, including several previously reported QTLs associated with cellulose synthase-like (Csl) genes, confirming the array’s effectiveness in trait dissection and genomic selection.

Across all currently published studies, the USDA-SoyWheOatBar-3K array consistently provided reliable genotype calls, broad genome coverage, and strong marker-trait associations, validating its application for high-throughput genomic studies in wheat, barley, and oat.

## Discussion

The small-grain component of the USDA-SoyWheOatBar-3K array was developed to provide standardized multi-species genotyping with informative genome-wide coverage for wheat, barley, and oat. The small-grain component evaluated here contained approximately 3,000 markers per crop and produced reproducible genotype calls across diverse germplasm and hybridization configurations. The platform is intended for routine breeding or fingerprinting applications rather than as a substitute for higher-density resources used for variant discovery or fine mapping.

### Species-specific performance and implications

The USDA-SoyWheOatBar-3K array demonstrated strong overall technical performance, although the types and frequency of problematic SNPs differed among crops. As expected, diploid barley had fewer problematic probes than polyploid wheat and oat. The principal issues involved ambiguous calls, absent amplification, reduced cluster separation, and excess heterozygous calls. Detailed analyses of cluster geometry, NULL alleles, and locus-specific failure modes compared to the available genomic resources are beyond the scope of this platform paper and will be reported separately.

Among the three species, barley exhibited the highest proportion of SNPs meeting all technical quality thresholds, consistent with its diploid genome structure and the selection of most barley SNPs from an established Illumina array (Bayer et al., 2017). With fewer homeologous regions compared to the hexaploid wheat and oat genomes, SNP clustering in barley was more straightforward, resulting in lower missing data rates and fewer problematic markers. The array’s performance in barley was further reinforced by the high compatibility of barley SNPs in multi-species hybridization settings (Keeble-Gagnère et al., 2021). These results indicate that the 3K array is well suited to barley breeding applications requiring accurate and rapid genotyping.

Probes for the polyploid crops also performed well on the USDA-SoyWheOatBar-3K array. Wheat probes showed 97.6% homozygote concordance with the original exome-capture genotype calls. However, wheat SNPs exhibited greater variability in technical performance, including greater within-cluster Theta variability and more calls in an artifactual heterozygous state. Oat SNPs were selected from existing Illumina-based and sequencing-based datasets. Across the evaluated source-platform comparisons, oat SNPs showed 92.1% homozygote concordance with the original genotypes. Oat SNPs also exhibited greater cluster variability than barley, including 13 SNPs with cluster-separation issues (Table 1). These comparisons highlight a common challenge in genotyping polyploids: the presence of three homoeologous subgenomes (A, B, and D in wheat; A, C, and D in oat) can reduce SNP specificity and increase Theta variability and missing-data rates (Rimbert et al., 2018; Tinker et al., 2014). The decline in oat concordance below 5% MAF likely reflects the sparse representation of minor-allele genotype classes, which makes both cluster definition and concordance estimates more sensitive to individual calling discrepancies between the source and array datasets. Homeologous probe binding and cluster compression in hexaploid oat may further accentuate this sensitivity. These mechanisms were not tested directly and are therefore presented as hypotheses.

Elevated heterozygosity in probe-based genotyping of polyploid genomes is a common challenge (Allen et al., 2017; Tinker et al., 2014; Wang et al., 2014). In our validation panels, heterozygous calls were most prevalent in oat (4.1% of SNPs), followed by barley (0.7%) and wheat (0.5%). Because cluster positions can vary among germplasm groups, a homozygous alternate allele confined to one group may fall near the heterozygous cluster when diverse panels are analyzed together, inflating apparent heterozygosity. For routine use in GenomeStudio or comparable clustering software, breeders should inspect loci showing excess heterozygosity, use species- and population-appropriate cluster files, and compare suspected heterozygous calls with parental or sequence-based genotypes when available. Cluster boundaries should be adjusted only when supported by reference genotypes and clear intensity separation; otherwise, ambiguous calls should be set to missing and persistently problematic assays should be excluded or replaced with locus-specific probes.

These species-specific insights provide valuable guidance for optimizing the use of the 3K array in breeding programs. While barley emerges as the most technically stable species for genotyping, wheat and oat require more careful consideration of hybridization configurations and SNP selection strategies. These findings are relevant to genomic selection and marker-assisted breeding pipelines because species-specific SNP behavior influences downstream genotype quality. Accounting for these differences can help breeders select appropriate hybridization configurations while maintaining reliable genotype calls.

### Impact of hybridization mode on data quality

Multi-species hybridization is a practical feature of the USDA-SoyWheOatBar-3K array because it allows samples from different crops to be processed in the same assay. Previous work with the Wheat Barley 40K SNP array demonstrated that joint hybridization can retain high genotype-call accuracy when probes are designed to target species-specific, predominantly single-copy loci (Keeble-Gagnère et al., 2021). However, cross-species probe binding, variation in DNA input, polymorphisms within probe-binding sequences, and hybridization to homologous or paralogous loci can shift cluster positions and reduce allele-cluster separation (Wang et al., 2014).

Multi-species effects were species- and partner-specific: no configuration was uniformly optimal across crops, and AB clusters were generally more sensitive than AA or BB clusters. These patterns indicate that adding genomic DNA from another crop altered fluorescence signals in a locus- and partner-dependent manner rather than causing a uniform reduction in signal quality.

Oat illustrates this partner dependence most clearly. Tri-hybridization produced the highest mean quality score for oat (0.893), although this was only marginally higher than the oat-wheat dual configuration (0.886), while oat-barley dual hybridization was lower (0.827). Because these comparisons may also reflect the specific genotypes represented in each configuration, the results should be interpreted as panel-specific rather than as evidence of an intrinsic oat advantage under tri-hybridization.

These partner-specific patterns do not support a simple explanation based solely on phylogenetic relatedness. Although wheat and barley belong to the Triticeae tribe, whereas oat belongs to the Aveneae tribe, hybridization performance likely reflects the combined effects of 50 bp probe-level sequence similarity, target-locus copy number, genome complexity, DNA input ratios, and SNP-specific cluster geometry (Keeble-Gagnère et al., 2021; Peng et al., 2022; Wang et al., 2014). Importantly, genotype concordance remained high across all configurations, ranging from 93.5% to 98.7%, and even SNPs classified as Poor retained concordance of 94.0%–99.1%. Thus, signal displacement and genotype-call failure represent related but distinct outcomes, and both should be considered when selecting markers for multi-species operation. Dual- and tri-hybridization can increase assay throughput and reduce the array-processing cost per sample, although these benefits must be weighed against the loss of some markers and the additional coordination and pooling required for multi-species submissions. Future versions of the array should prioritize probes that target unique sequences within the intended genome and have minimal similarity to potential binding sites in the other crop genomes.

### Independent validation studies

Independent studies in wheat and oat support the utility of the USDA-SoyWheOatBar-3K array for breeding applications. In spring wheat, Brault et al. (2025) showed that approximately 2,000 retained 3K markers supported moderate-to-high genomic prediction of Fusarium head blight and agronomic traits, with training-population size having a greater effect than further increases in marker density. Gill et al. (2026) subsequently used SNP-array and targeted KASP genotyping to characterize genetic diversity and the distribution of Rht-B1b, Rht-D1b, Rht24, and Rht25 across 1,013 North American hard red spring wheat lines representing approximately a century of breeding. In oat, Dhakal et al. (2024) incorporated 3K-array genotypes into multi-trait genomic prediction for milling-quality traits, while Bazzer et al. (2025) found that the 3 K array produced prediction accuracies comparable to GBS and supported multi-environment GWAS for beta-glucan content. Together, these studies demonstrate that the array provides sufficient genome-wide information for genomic prediction, association analysis, and historical germplasm characterization at substantially lower marker density than high-density platforms.

### Implications for breeding programs

High-throughput genotyping supports genomic selection and marker-assisted selection when it provides reproducible marker data at a density appropriate for downstream breeding applications (Bhat et al., 2016). The USDA-SoyWheOatBar-3K array was designed for routine breeding populations in which standardized marker recovery and sufficient linkage-disequilibrium coverage are more important than maximal marker density. Its fixed, evenly distributed marker set reduces dependence on cycle-specific locus recovery, while independent applications indicate that the retained density is sufficient for genomic prediction and diversity analysis. This positioning is consistent with the broader move toward public mid-density genotyping resources for operational breeding, where reproducibility and per-sample cost weigh more heavily than marker number (Dreisigacker et al., 2026). Higher-density arrays or sequencing remain preferable for fine mapping and variant discovery.

In breeding applications, genomic selection relies on genome-wide markers in linkage disequilibrium (LD) with causal variants (Heffner et al., 2009). The 3K marker set was distributed across genetic-map intervals to provide broad genome coverage and support genomic prediction across breeding populations.

The USDA-SoyWheOatBar-3K array integrates known trait-linked loci with genome-wide tagging markers. Of the 9,468 small-grain SNPs, 490 were designated for MAS, comprising 94 barley, 165 oat, and 231 wheat markers (Supplementary Table S2). These markers represent disease resistance, phenology, grain quality, yield-related traits, and abiotic-stress responses. Their inclusion enables major-effect loci to be tracked using the same assay employed for genomic prediction, relatedness assessment, and population analysis. However, the utility of an individual MAS marker remains dependent on its validation and linkage phase within the target germplasm. The USDA-SoyWheOatBar-3K array is available through Illumina. Researchers interested in current pricing or genotyping services through the Small Grains Genotyping Laboratory in Fargo, North Dakota, may contact the corresponding author.

## Conclusion

The small-grain component of the USDA-SoyWheOatBar-3K array provides a reproducible, breeding-oriented genotyping platform for wheat, barley, and oat. Across the three crops, the array retained high-quality SNP performance and strong genotype concordance, while the multi-species hybridization analyses demonstrated that dual- and tri-hybridization can further increase assay efficiency without substantially compromising genotype-call accuracy. The partner-specific differences observed in signal quality also emphasize the importance of probe specificity and crop-specific genome complexity when implementing multi-species genotyping.

Although the relatively low marker density is not intended to capture all genetic variation within these large and diverse genomes, the array provides sufficient genome-wide coverage for routine applications including genomic prediction, association analysis, germplasm characterization, and marker-assisted breeding, as demonstrated by both the present study and independent applications of the platform. The fixed marker content additionally provides a standardized framework for comparing germplasm across breeding programs, environments, and generations. Imputation to increase marker density was not evaluated in the present study but remains a future opportunity with appropriate populations for empirical testing (Marchini and Howie, 2010). Overall, the USDA-SoyWheOatBar-3K array offers a practical balance of marker density, reproducibility, and throughput for routine genomics-assisted breeding across the major U.S. small-grain crops.

## Data Availability

The original contributions presented in the study are included in the article/Supplementary Material, further inquiries can be directed to the corresponding author.
